# Rapid and Accurate Multiple Testing Correction and Power Estimation for Millions of Correlated Markers

**DOI:** 10.1371/journal.pgen.1000456

**Published:** 2009-04-17

**Authors:** Buhm Han, Hyun Min Kang, Eleazar Eskin

**Affiliations:** 1 Department of Computer Science and Engineering, University of California San Diego, La Jolla, California, United States of America; 2 Department of Computer Science, University of California Los Angeles, Los Angeles, California, United States of America; 3 Department of Human Genetics, University of California Los Angeles, Los Angeles, California, United States of America; Princeton University, United States of America

## Abstract

With the development of high-throughput sequencing and genotyping technologies, the number of markers collected in genetic association studies is growing rapidly, increasing the importance of methods for correcting for multiple hypothesis testing. The permutation test is widely considered the gold standard for accurate multiple testing correction, but it is often computationally impractical for these large datasets. Recently, several studies proposed efficient alternative approaches to the permutation test based on the multivariate normal distribution (MVN). However, they cannot accurately correct for multiple testing in genome-wide association studies for two reasons. First, these methods require partitioning of the genome into many disjoint blocks and ignore all correlations between markers from different blocks. Second, the true null distribution of the test statistic often fails to follow the asymptotic distribution at the tails of the distribution. We propose an accurate and efficient method for multiple testing correction in genome-wide association studies—SLIDE. Our method accounts for all correlation within a sliding window and corrects for the departure of the true null distribution of the statistic from the asymptotic distribution. In simulations using the Wellcome Trust Case Control Consortium data, the error rate of SLIDE's corrected *p*-values is more than 20 times smaller than the error rate of the previous MVN-based methods' corrected *p*-values, while SLIDE is orders of magnitude faster than the permutation test and other competing methods. We also extend the MVN framework to the problem of estimating the statistical power of an association study with correlated markers and propose an efficient and accurate power estimation method SLIP. SLIP and SLIDE are available at http://slide.cs.ucla.edu.

## Author Summary

In genome-wide association studies, it is important to account for the fact that a large number of genetic variants are tested in order to adequately control for false positives. The simplest way to correct for multiple hypothesis testing is the Bonferroni correction, which multiplies the *p*-values by the number of markers assuming the markers are independent. Since the markers are correlated due to linkage disequilibrium, this approach leads to a conservative estimate of false positives, thus adversely affecting statistical power. The permutation test is considered the gold standard for accurate multiple testing correction, but is often computationally impractical for large association studies. We propose a method that efficiently and accurately corrects for multiple hypotheses in genome-wide association studies by fully accounting for the local correlation structure between markers. Our method also corrects for the departure of the true distribution of test statistics from the asymptotic distribution, which dramatically improves the accuracy, particularly when many rare variants are included in the tests. Our method shows a near identical accuracy to permutation and shows greater computational efficiency than previously suggested methods. We also provide a method to accurately and efficiently estimate the statistical power of genome-wide association studies.

## Introduction

Association studies have emerged as a powerful tool for discovering the genetic basis of human diseases [1]–[3]. With the development of sequencing and high-throughput genotyping technologies, the number of single nucleotide polymorphism (SNP) markers genotyped by current association studies is dramatically increasing. The large number of correlated markers brings to the forefront the multiple hypothesis testing correction problem and has motivated much recent activity to address it [4]–[6].

There are two common versions of the multiple testing correction problem: per-marker threshold estimation and p-value correction. In a typical study which collects 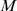 markers, at each marker, we perform a statistical test and obtain a p-value which we refer to as a *pointwise p-value*. We would like to know how significant a pointwise p-value needs to be in order to obtain a significant result given that we are observing 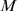 markers. The *per-marker threshold* can be defined as the threshold for pointwise p-values which controls the probability of one or more false positives [6]. Similarly, we would like to quantitatively measure the significance of a pointwise p-value taking into account that we are observing 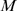 markers. For each pointwise p-value, the *corrected p-value* can be defined as the probability that, under the null hypothesis, a p-value equal to or smaller than the pointwise p-value will be observed at any marker [7]. For example, the Bonferroni correction corrects a pointwise p-value 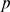 to 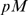, or estimates the per-marker threshold as 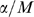 given a significance threshold 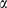.

While the Bonferroni (or Šidák) correction provides the simplest way to correct for multiple testing by assuming independence between markers, permutation testing is widely considered the gold standard for accurately correcting for multiple testing [7]. However, permutation is often computationally intensive for large data sets [4]. For example, running 1 million permutations for a dataset of 500,000 SNPs over 5,000 samples takes up to 4 CPU years using widely used software such as PLINK [8] (See Results). On the other hand, the Bonferroni (or Šidák) correction ignores correlation between markers and leads to an overly conservative correction, which is exacerbated as the marker density increases.

**Figure 1:**
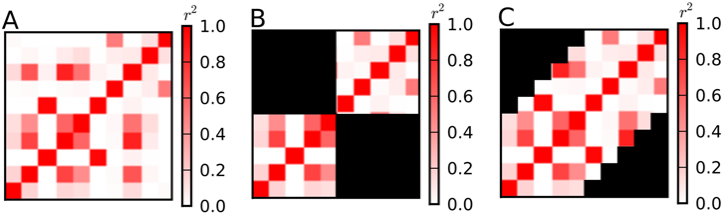
Block-wise strategy and sliding-window approach. (A) Correlations between 10 markers are depicted. (B) Correlations taken into account by a block-wise strategy with a block size of 5. The ignored correlations are shown as black. (C) Correlations taken into account by a sliding-window approach with a window size of 5. The ignored correlations are shown as black.

In this paper, we correct for multiple testing using the framework of the multivariate normal distribution (MVN). For many widely used statistical tests, the statistics over multiple markers asymptotically follow a MVN [9],[10]. Using this observation, several recent studies [4],[9],[10] proposed efficient alternative approaches to the permutation test, and showed that they are as accurate as the permutation test for small regions at the size of candidate gene studies (with <1% average error in corrected p-values) [4]. However, when applied to genome-wide datasets, they are not as accurate. In our analysis of the Wellcome Trust Case Control Consortium (WTCCC) data [11], these methods eliminate only two-thirds of the error in the corrected p-values relative to the Bonferroni correction. There are two main reasons why these methods do not eliminate all of the error. First, the previous MVN-based methods can be extended to genome-wide analyses only by partitioning the genome into small linkage disequilibrium (LD) blocks and assuming markers in different blocks are independent, because they can handle only up to hundreds of markers in practice [4],[9]. This block-wise strategy leads to conservative estimates because inter-block correlations are ignored (Figure 1B). Second, these methods do not account for the previously unrecognized phenomenon that the true null distribution of a test statistic often fails to follow the asymptotic distribution at the extreme tails of the distribution, even with thousands of samples.

We propose a method for multiple testing correction called SLIDE (a **S**liding-window approach for **L**ocally **I**nter-correlated markers with asymptotic **D**istribution **E**rrors corrected), which differs from previous methods in two aspects. First, SLIDE uses a sliding-window approach instead of the block-wise strategy. SLIDE approximates the correlation matrix as a band matrix (a matrix with non-zero elements along the diagonal band), which can effectively characterize the overall correlation structure between markers given a sufficiently large bandwidth. Then SLIDE uses a sliding-window Monte-Carlo approach which samples a statistic at each marker by conditioning on the statistics at previous markers within the window, accounting for entire correlation in the band matrix (Figure 1C).

Second, SLIDE takes into account the phenomenon that the true null distribution of a test statistic often fails to follow the asymptotic distribution at the tails of the distribution. It is well known that if the sample size is small, the true distribution and the asymptotic distribution show a discrepancy [12],[13]. However, to the best of our knowledge, the effect of this discrepancy in the context of association studies has not been recognized, since thousands of samples are typically not considered a small sample. We observe that this discrepancy often appears in genome-wide association studies, even with thousands of samples, because of the extremely small genome-wide per-marker threshold (or pointwise p-value). The error caused by this discrepancy is more serious for datasets with a large number of rare variants, highlighting the importance of this problem for association studies based on next-generation sequencing technologies (See Materials and Methods). SLIDE corrects for this error by scaling the asymptotic distribution to fit to the true distribution.

With these two advances, SLIDE is as accurate as the permutation test. In our simulation using the WTCCC dataset [11], the error rate of SLIDE's corrected p-values is more than 20 times smaller than the error rate of previous MVN-based methods' corrected p-values, and 80 times smaller than the error rate of the Bonferroni-corrected p-values. Our simulation using the 2.7 million HapMap SNPs [14] shows that SLIDE is accurate for higher-density marker datasets as well. In contrast, the error rates of previous MVN-based methods increase with the marker density, since the dataset will include more rare variants. Computationally, our simulation shows that SLIDE is orders of magnitude faster than the permutation test and faster than other competing methods.

The MVN framework for multiple testing correction is very general, allowing it to be applied to many different contexts such as quantitative trait mapping or multiple disease models [4]. We show that the MVN framework can also correct for multiple testing for the weighted haplotype test [15],[16] and the test for imputed genotypes based on the posterior probabilities [17].

In addition to multiple testing correction, we extend the MVN framework to solve the problem of estimating the statistical power of an association study with correlated markers. There are two traditional approaches to this problem: a simulation approach constructing case/control panels from the reference dataset [4],[10],[17],[18], which is widely considered the standard but is computationally intensive; and the best-tag Bonferroni method [19]–[21], which is an efficient approximation but is often inaccurate.

The power estimation problem can be solved within the MVN framework because the test statistic under the alternative hypothesis follows a MVN centered at the non-centrality parameters (NCP). The vector of the NCPs turns out to be approximately proportional to the vector of correlation coefficients (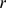) between the causal SNP and the markers. This is a multi-marker generalization of the Pritchard and Preworzki [22] single-marker derivation of the NCP proportional to 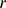. Our method SLIP (**S**liding-window approach for **L**ocally **I**nter-correlated markers for **P**ower estimation) efficiently estimates a study's power using the MVN framework.

Seaman and Müller-Myhsok [9] and Lin [10] pioneered the use of the MVN for multiple testing correction. Seaman and Müller-Myhsok described the direct simulation approach (DSA) method. Conneely and Boehnke [4] increased its efficiency by adapting an available software package called mvtnorm [23],[24]. Both studies primarily focused on datasets used in candidate gene studies and suggested the block-wise strategy as a possible approach for genome-wide studies.

Another approach for multiple testing correction is to estimate the effective number of tests from eigenvalues of the correlation matrix [25]–[27]. Recently, Moskvina and Schmidt [6] and Pe'er *et al.* [28] showed that the effective number of tests varies by the p-value levels, demonstrating that a method estimating a constant effective number can be inaccurate. Moskvina and Schmidt [6] proposed a pairwise correlation-based method called Keffective, which estimates the effective number taking into account the significance level. Keffective is a sliding-window approach similar to SLIDE, but it differs because within each window it uses the pairwise correlation to the most correlated marker, while SLIDE uses the conditional distribution given all markers. Fitting the minimum p-value distribution by a beta distribution [29] has been shown often to be inaccurate [6]. Kimmel and Shamir [30] developed an importance sampling procedure called rapid association test (RAT). RAT is efficient for correcting very significant p-values, but requires phased haplotype data.

Connecting the multiple testing correction and power estimation problems leads to the insight that the per-marker threshold estimated from the reference dataset for estimating power can be used as a precomputed approximation to the true per-marker threshold for the collected samples. In simulations using the WTCCC control data, we show that the per-marker threshold estimated from the HapMap CEU population data approximately controls the false positive rate.

Our methods SLIP and SLIDE require only summary statistics such as the correlation between markers within the window size, allele frequencies, and the number of individuals. Therefore unlike the permutation test, our method can still be applied even if the actual genotype data is not accessible. Our methods are available at http://slide.cs.ucla.edu.

## Materials and Methods

### Multiple Testing Correction

#### Multivariate normal approximation

For many widely used statistical tests, the vector of statistics over multiple markers asymptotically follows a MVN [9],[10]. The covariance matrix of the MVN can be derived for many popular statistical tests such as Armitage's trend test in the context of the general score test [4],[9]. We perform this derivation at the haplotype level using the properties of the hypergeometric distribution in the context of the 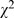 test in order to highlight the connection between the multiple testing correction and the power estimation problems. In Text S1, we also derive the covariance for the weighted haplotype test [15],[16] and the test for imputed genotypes [17],[31],[32]. All of the results presented here for balanced case/control studies can be extended to unbalanced studies. We will interchangeably use the terms ‘covariance matrix’ and ‘correlation matrix’, because the variances are 1.

Assume we permute 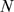 case haplotypes and 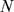 control haplotypes. Let 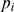 be the minor allele frequency (MAF) at marker 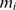 estimated from the sample. Let 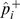 and 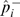 be the observed MAFs in the permuted case and control haplotypes. Although 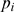 itself is an observed value from the sample, we will consider it as a constant because it is invariant over random permutations. The minor allele count in the permuted case haplotypes, 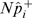, follows a hypergeometric distribution. If 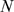 is large, the test statistic at 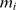


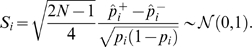
The squared statistic differs from the Pearson's 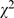 statistic by a constant 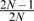.

Let 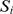 and 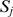 be the statistics at marker 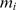 and 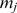. Let 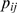, 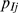, 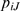, 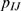 be the sample frequencies of the four haplotypes with minor and major alleles at 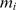 and 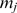 respectively. A random permutation is equivalent to selecting 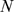 case haplotypes from 4 bins of different haplotypes. Thus, the haplotype count in the permuted case haplotypes, (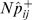, 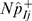, 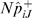, 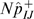), follows a multivariate hypergeometric distribution. By the properties of the hypergeometric distribution,
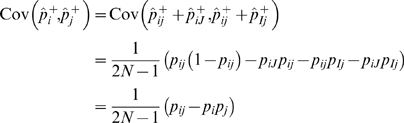


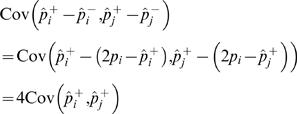
(1)

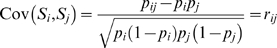
(2)where 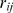 is the correlation coefficient between 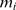 and 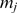 measured in the sample.

**Figure 2:**
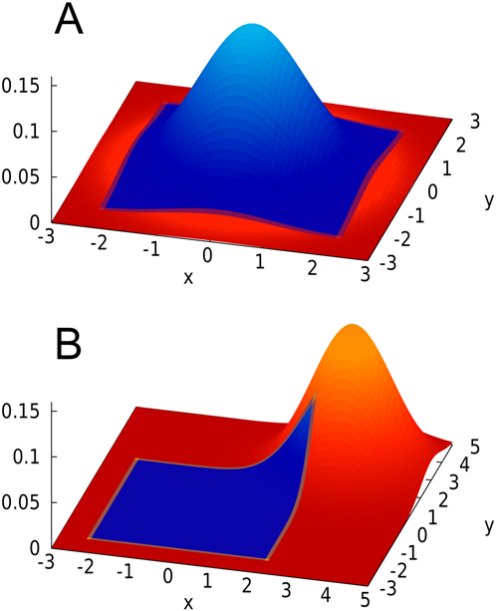
Probability density function of a bivariate MVN at two markers. The area outside the rectangle is the critical region. (A) Under the null hypothesis, the MVN is centered at zero. The outside-rectangle probability is the corrected p-value (or the significance level). (B) Under the alternative hypothesis, the MVN is shifted by the non-centrality parameter. The outside-rectangle probability is power.

Let 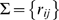 be the 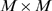 covariance matrix between 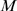 markers. By the multivariate central limit theorem [33], if 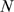 is large, the vector of statistics 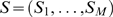 asymptotically follows a MVN with mean zero and variance 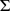. Given a pointwise p-value 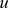, let 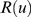 be the 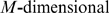 rectangle with corners 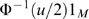 and 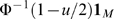 where 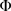 is the cumulative density function (c.d.f.) of the standard normal distribution and 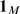 is the vector of 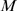 ones. The corrected p-value 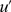 is approximated as the outside-rectangle probability,
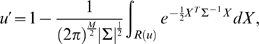
(3)as shown in Figure 2A. Similarly, given a significance threshold 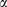, the per-marker threshold 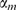 is approximated by searching for a pointwise p-value whose corrected p-value is 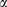.

#### Discrepancy between asymptotic and true distributions

If the asymptotic MVN closely approximates the true distribution of the statistic, then Formula (3) will provide an accurate multiple testing correction; this has been shown to be true for small regions such as those tested in candidate gene studies [4]. One may expect that the discrepancy between the asymptotic and true distributions would be negligible in current association studies, given their thousands of samples.

However, we observe that this discrepancy can appear in genome-wide association studies, in spite of the large sample size, because of the extremely small per-marker threshold (or pointwise p-value) caused by the large number of tests. At its extreme tails, the asymptotic distribution is typically thicker than the true distribution.

**Figure 3:**
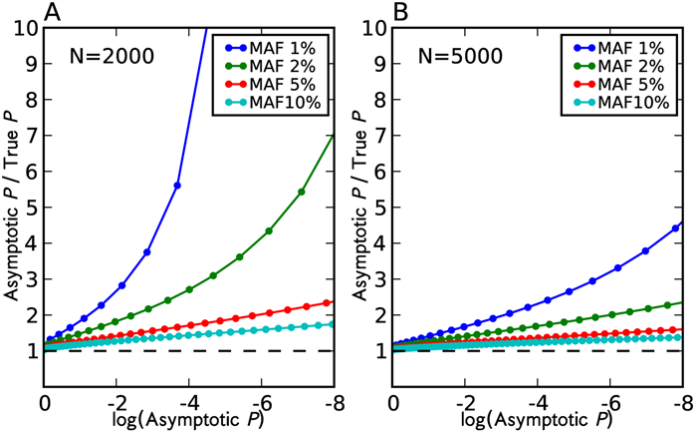
Discrepancy between asymptotic p-value and true p-value in a single SNP experiment. Given a 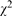 threshold 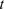, the asymptotic p-value is 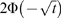. The true p-value is obtained by listing all possible contingency tables. The number of individuals (N) denotes the number of haplotypes, half control and half case.

This phenomenon can be illustrated with a single-SNP experiment using the 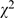 test. For a threshold 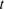, the asymptotically approximated p-value (asymptotic p-value) is 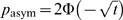. Assume 1,000 case and 1,000 control haplotypes. Given a fixed number of minor alleles, we can list every possible 2×2 table. The true p-value 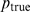 is the sum of the probabilities of the tables whose statistic is 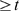. If the asymptotic approximation is accurate, then 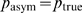. We compare these two p-values for many different thresholds and plot the ratio in Figure 3. We repeat the experiments for various MAFs and sample sizes.

Figure 3 shows that even with thousands of samples, at the genome-wide significance level, the asymptotic p-value is highly inflated compared to the true p-value. The inflation is more dramatic for SNPs with small MAFs. We observe the similar phenomenon using genotypes and the trend test (data not shown).

One may argue that this phenomenon is not important because it mostly occurs at rare SNPs (MAF≤5%) where current studies already have low power to detect associations. However, an incorrect approximation of the distributions at some SNPs affects the corrected p-values of all SNPs. This is because the corrected p-value depends on the distributions of the statistics at all of the SNPs, as it is defined as the probability observing significant results at any marker. For example, suppose we approximate 10 independent normal distributions at 10 independent SNPs. Assume that we correctly approximate 9 distributions, but for one distribution we think that the tails are thicker than the true distribution by a factor of 100. For any given pointwise p-value 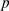, the true corrected p-value is 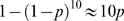 by the Šidák correction. However, we will estimate the corrected p-value as 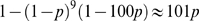 by integrating over the MVN. This shows that incorrectly approximating the distributions at rare SNPs can adversely affect the corrected p-values of all SNPs, including common SNPs.

One can avoid this type of error in corrected p-values by using a method not dependent on the asymptotic approximation, such as the permutation test, or by eliminating rare SNPs in the analysis. It may be sensible to remove rare SNPs with a few or tens of minor allele counts, if the power is very low or if the SNPs are error-prone in their calling. However, Figure 3 shows that the error caused by using the asymptotic approximation happens even at SNPs with minor allele counts in the hundreds. Therefore removing all of them will decrease our power to detect associations.

#### SLIDE

SLIDE corrects for multiple testing by using a sliding-window approach to approximate the MVN and then scaling the MVN to approximate the true distribution of the statistic. There are two underlying intuitions. First, a sliding window approach takes into account most of the correlations in the data due to the local LD structure. Second, even though the asymptotic MVN shows a departure from the true distribution at the tail, the scaled MVN will closely approximate the true distribution because the covariance between the statistics is identical in both the true distribution and the MVN. (The covariance derivation does not involve the central limit theorem.)

**Step 1** — SLIDE first approximates the MVN by using a sliding-window Monte-Carlo approach. Given 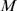 markers, let (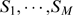) be the vector of statistics which asymptotically follows a MVN under the null hypothesis. Let 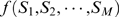 be the joint probability density function (p.d.f.) of the statistics. Our goal is to generate a large number of samples, (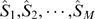), to approximate the MVN. If 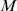 is very large, the standard sampling approach using the Cholesky decomposition [34] is impractical unless we split the region into small blocks.

Under the local LD assumption, the statistics at distant markers are uncorrelated. Thus, given a window size 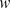, we can assume that 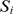 is conditionally independent of 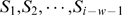 given 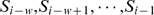. Then by the chain rule,
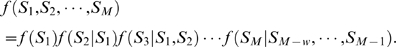
Thus, 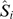 can be sampled given 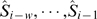, based on the conditional distribution 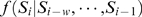. The conditional distributions are given by the standard formula for the MVN. Thus we can efficiently generate a large number of samples. The procedure is described in detail in Text S2.

**Figure 4:**
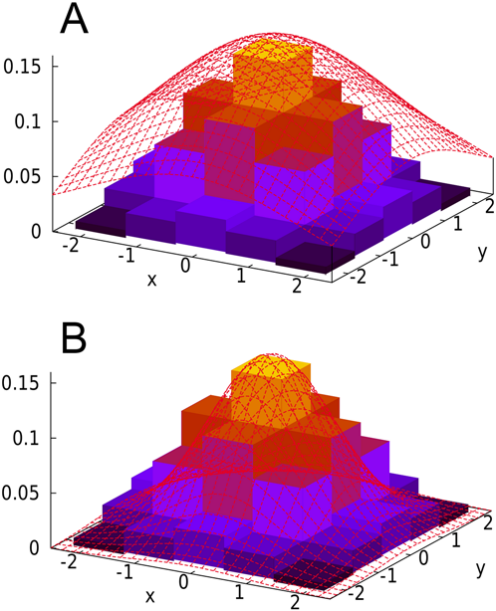
SLIDE's scaling procedure. The probability density function of the asymptotic bivariate MVN is depicted as a grid. The probability mass function of the true distribution is depicted as a histogram. (A) The asymptotic distribution often shows a discrepancy from the true distribution. (The discrepancy is exaggerated in this figure.) (B) After scaling down the asymptotic distribution, the discrepancy is removed.

**Step 2** — We scale the approximated MVN to fit to the true distribution of the statistic (Figure 4). The rationale for this step is that, if we only consider the marginal distribution at each marker, it is possible to analytically compute the true distribution by listing all possible 2×2 or 2×3 contingency tables [35]. This allows us to directly compare the asymptotic distribution and the true distribution, and to compute how much we should scale the asymptotic distribution to fit to the true distribution.

The level of discrepancy between the asymptotic and true distributions is large at the tails of the distribution compared to the center. Thus, in order to scale the asymptotic distribution to fit to the true distribution, we cannot multiply the entire distribution by a single scaling factor, but must instead compute the scaling factor for each different threshold.

Given a 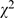 threshold 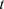, we compute the scaling factor as follows. The asymptotic p-value is 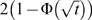. Let 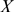 be a random variable following the true discrete distribution of the 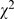 statistic. The exact true p-value is 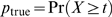. The scaling factor is computed as 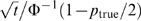, because if we scale the standard normal distribution by this factor, the asymptotic p-value for the scaled distribution becomes exactly 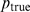 at the threshold 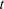. In practice, we find that using the so-called mid p-value 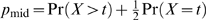 [35] instead of 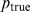 provides a better approximation to the true distribution.

Note that, for unbalanced case/control studies, the level of discrepancy is not symmetric at the upper and lower tails of the normal distribution. Thus, we should compute the scaling factor for each tail of the normal distribution separately.

**Step 3** — Given the scaled MVN, p-values are corrected by integrating over the outside of the rectangle as in Formula (3).

### Power Estimation

#### Assumptions

A discussion of association study power depends on many arbitrary assumptions. Though our framework can be extended to other assumptions, in this paper, we adopt those used in De Bakker *et al.* [18]: (1) The disease status is affected by a single SNP. (2) The allele effect is multiplicative. (3) The relative risk is known. (4) The phased reference dataset represents the population.(5) All marker SNPs are in the reference dataset. (6) All possible causal SNPs are in the reference dataset. (7) Each possible causal SNP is equally likely to be causal.

For complex diseases, assumption (1) can still be applied if each causal SNP marginally contributes to the risk. Assumptions (4) and (5) can lead to an overestimation of power, especially if the markers are chosen using the reference dataset [36]. Instead of assumption (7), a non-uniform distribution can also be used [37].

Finally, we assume that the investigator has determined the number of individuals in the study and the significance threshold.

#### Multivariate normal approximation

We extend the MVN framework to the power estimation problem. Consider a study design which defines markers and plans to collect 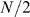 case and 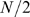 control diploid individuals. Let 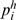 be the population MAF at marker 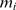 estimated from the reference dataset (‘h’ denoting the HapMap [14]). Let 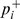 and 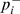 be the MAFs in the case and control populations.

**Single marker** — If marker 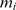 is causal for a disease of prevalence 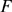 with relative risk 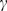, under the multiplicative model,
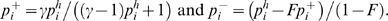
(4)


The case/control study can be thought of as a procedure which draws 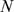 chromosomes from the case population and 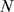 chromosomes from the control population. Let 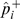 and 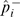 be random variables denoting the observed MAFs in the collected cases and controls. Let 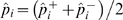 and 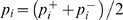. Then, since each of 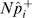 and 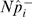 follows a binomial distribution, if 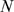 is large, the test statistic at marker 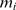


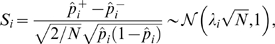
where
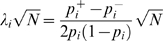
is the non-centrality parameter.

If the marker and the causal SNP are distinct (a condition called *indirect association*), the NCP derivation changes. Suppose a SNP 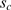 is causal but we collect marker 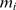. If we put an imaginary marker 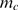 at SNP 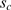, we can compute the NCP at marker 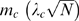, and compute the correlation coefficient between 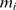 and 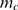 from the reference dataset (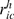). Pritchard and Preworzki [22] show that the NCP at marker 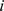 is approximately 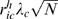.

**Multiple markers** — We examine the covariance between the statistic 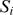 at marker 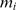 and 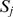 at marker 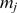 given that SNP 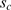 is causal. Let 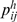, 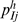, 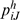, 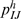 be the haplotype frequencies with minor and major alleles at 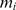 and 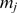 respectively, in the overall population. Let 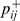, 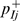, 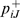, 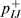 and 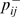, 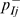, 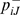, 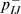 be the frequencies in the case and control populations.

Collecting cases (or controls) is equivalent to drawing 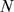 chromosomes from four possible haplotypes. Thus, the haplotype count in cases, (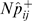, 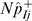, 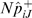, 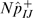), follows a multinomial distribution. By the properties of the multinomial distribution,
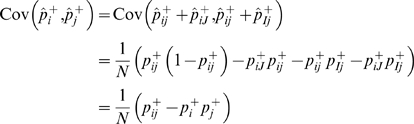


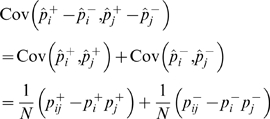


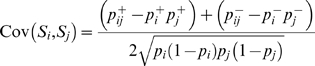
(5)

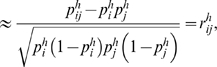
(6)where 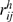 is the correlation coefficient between 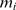 and 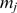 estimated from the reference dataset.

In practice, approximation in Formula (6) usually leads to an accurate power estimate. However, if the relative risk is very large, the Formula (5) can be computed exactly and used as follows. By Formula (4), we can calculate 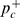 and 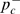, the MAFs of the causal SNP 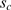 in the case and control populations. We can then estimate 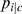 or 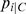, the conditional probability that we will observe the minor allele at 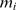 given we observe the minor or major allele at 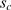. Note that these conditional probabilities are exactly, not approximately, invariant between cases and controls (See Text S3). Therefore 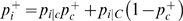. We can similarly estimate 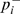 and the haplotype frequencies (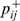 and 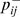), which allows us to compute Formula (5).

Let 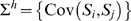 be the 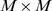 covariance matrix between 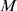 markers. Let
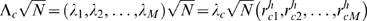
(7)be the vector of NCPs induced by the causal SNP 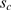. By the multivariate central limit theorem [33], if 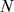 is large, the vector of statistics (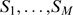) asymptotically follows a MVN with mean 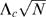 and variance 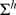.

Power depends on the per-marker threshold 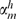. Given a significance threshold 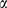, 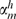 is set to a level which controls the outside-rectangle probability of the null MVN at 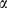 such that
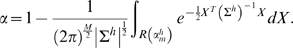
(8)


Given 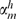, the per-causal-SNP power with respect to a causal SNP 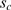 is the outside-rectangle probability of the alternative MVN,
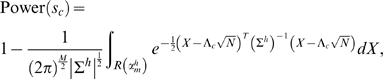
(9)as shown in Figure 2B. The average power is obtained by averaging per-causal-SNP powers over all putative causal SNPs.

#### SLIP

Our method SLIP estimates the power of a study design using the MVN framework. First, like SLIDE, SLIP estimates the per-marker threshold in Formula (8) using a sliding window approach. Then SLIP samples causal SNPs, approximates the alternative MVN to estimate the per-causal-SNP power, and averages per-causal-SNP powers over sampled causal SNPs.

Since power is typically larger (e.g. 80%) than a p-value (e.g. .01), a small error in the per-marker threshold barely affects the estimate. Thus, the error caused by using the asymptotic approximation is negligible. Also, given a causal SNP, we can assume that nearby markers (e.g. those within ±1 Mb) can capture most of the statistical power due to local LD. Thus, we can set a window size and only use the markers within that window to estimate the alternative MVN, which will be a 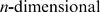 marginal MVN if we use 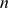 markers.

The computation becomes very efficient if we use approximation (6). Since approximation (6) states that the covariance is the same for the null and alternative MVNs, we can re-use the null MVN constructed for estimating the per-marker threshold, by shifting it by the NCP to get the alternative MVN. If we re-use the random samples this way, the constructed random samples will be not completely random, as they depend on each other. However, we observe that the inaccuracy caused by this dependency is negligible if we generate a large number of samples for the null MVN. If we re-use the samples, then with almost no additional computational cost, SLIP can generate power estimates for multiple relative risks or study sample sizes, since these only change the NCP.

### Multiple Testing Correction Using Reference Dataset

Multiple testing correction is generally performed using the collected data and not the reference data. Recall that the difference between the per-marker threshold for multiple testing correction (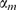) and the per-marker threshold for power estimation (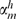) is that the former is estimated from the collected data, the latter from the reference data. We suggest that multiple testing can be approximately corrected using the reference data, by using 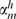 as a substitute of 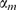. The advantage is that we can obtain an idea of the per-marker threshold even before the samples are collected. In Results, we show the accuracy of this approximation using the HapMap data and the WTCCC data.

### Genotype Data

We downloaded the HapMap genotype data (release 23a, NCBI build 36) from the HapMap project web site [14],[38] and phased the data into haplotypes using HAP [39], which can handle the trio information. We downloaded the case/control genotype data from the Wellcome Trust Case Control Consortium web site [11] and phased it into haplotypes using Beagle [40].

### Web Resources

The URL for methods presented herein is as follows: http://slide.cs.ucla.edu

## Results

### Multiple Testing Correction

#### P-value correction in Chromosome 22 of WTCCC data

In order to compare how accurately and efficiently different methods correct multiple testing, we simulate a study using the WTCCC data [11]. We use the chromosome 22 data (5,563 SNPs) of the Type 2 diabetes (T2D) case/control study (4,862 individuals). Since not every method can be applied to unphased genotype data, we use haplotype data using the allelic 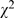 test and permutation by chromosomes. We first remove any existing associations by randomly dividing the chromosomes into half cases and half controls. Removing associations is necessary because to correct a pointwise p-value, RAT currently requires an actual SNP with that pointwise p-value to be implanted in the dataset as the most significant SNP.

First, we perform 10 M permutations to correct ten different pointwise p-values from 10^−4^ to 10^−7^, whose corrected p-values are from .04 to .0004. We will consider the corrected p-values by the permutation test as the gold standard, and call them *permutation p-values*. We will assume a method is accurate if its corrected p-values are close to the permutation p-values.

We use SLIDE, DSA, mvtnorm, RAT, and Keffective to correct p-values. DSA and mvtnorm are MVN-based methods using the block-wise strategy. We use a constant block size (window size) of 100 markers for all methods. Since RAT defines the window size in terms of physical distance, we use 600 kb, the average distance of 100 markers in the dataset. We use -X -e2 option for RAT for an exact computation of the importance sampling procedure as suggested by Kimmel and Shamir [30]. For every method, we use a large number (>1 M) of sampling iterations, which allows 95% confidence interval within ±.01*p* for 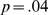 and ±.1*p* for 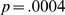. Keffective corrects p-values by estimating the effective number of tests for a significance threshold and dividing the pointwise p-values by that number. We use 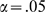 and window size of 100 for Keffective.

**Figure 5:**
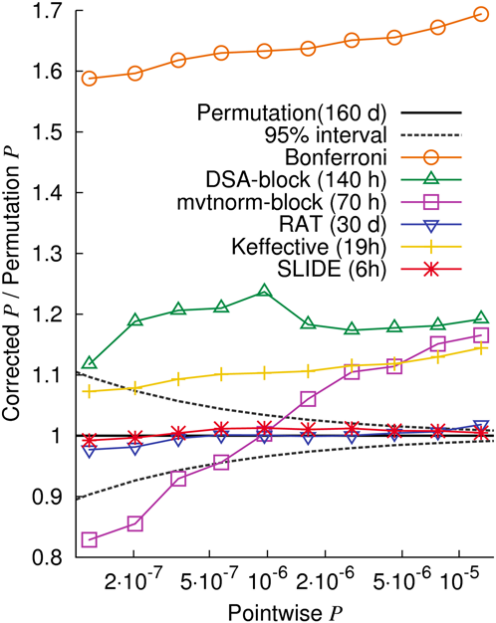
Ratios between corrected p-values and permutation p-values for ten different p-value thresholds. We use the WTCCC T2D case/control chromosome 22 data. Approximated time is for correcting 10 p-values with respect to 500 K SNPs assuming 100 K permutations. The dashed lines denote the interval where an accurate methods' estimate will be found more than 95% of the time.

Figure 5 shows the ratios between the ten corrected p-values and the permutation p-values. An accurate method will yield a ratio of 1 for all ten different thresholds. The dashed lines denote the area where an accurate method's estimate will be found more than 95% of the time. As expected, the Bonferroni correction is very conservative, overestimating the p-values by 64% on average.

DSA is conservative with an average error of 19%. This is equivalent to reducing the error by only about two thirds relative to the Bonferroni correction. The reasons for the errors include the block-wise strategy ignoring inter-block correlations, and not correcting for the error caused by using the asymptotic approximation. In addition to these errors, mvtnorm suffers from an anti-conservative bias which grows as the p-value becomes more significant. This is because the p-value in each block is too small for mvtnorm to accurately estimate. Our simulation shows that this anti-conservative bias increases with the number of sampling iterations (data not shown).

Keffective is more accurate and faster than DSA and mvtnorm. The average error of Keffective is 10.6%. Note that Keffective is optimized to provide an efficient approximation for the effective number of tests within ∼10% of error. Thus, Keffective is achieving its goal.

Both RAT and SLIDE show accurate estimates with the same average error of 0.8%. Thus, the error rate of SLIDE's corrected p-values is more than 10 times smaller than the error rate of Keffective's corrected p-values, more than 20 times smaller than the the error rate of DSA's corrected p-values, and 80 times smaller than the error rate of the Bonferroni-corrected p-values.

We now explore how each source of error in MVN-based methods – the block-wise strategy and the use of the asymptotic approximation without correction – affects the error rate. We remove 1,048 rare SNPs (MAF<.05) and perform multiple testing correction with respect to the remaining 4,515 common SNPs. When considering only common SNPs, the error caused by using the asymptotic approximation will be much smaller (See Materials and Methods). Figure S1 shows that the average error of DSA is reduced from 19% to 3.5%, showing that a considerable amount of the error is due to using the asymptotic approximation without correction. The error of Keffective is also reduced from 10.6% to 6.5%. The error of mvtnorm is increased from 9.4% to 12.9% because the conservative error caused by using the asymptotic approximation no longer compensates for its anti-conservative bias. SLIDE and RAT are consistently accurate regardless of the exclusion of rare SNPs. Although many methods look relatively accurate when considering only common SNPs, they are inaccurate when considering all SNPs.

Table 1 shows the extrapolated running time of each method for correcting p-values with 500 K SNPs tested over 5,000 individuals. The running times of RAT, DSA, and mvtnorm increases linearly with the number of p-values we correct, since they are currently implemented to correct one p-value at a time (though this may change in future versions). Since Keffective is not a sampling approach, its running time is independent of the number of samples. Given a window size of 100, our time estimate for Keffective (19 h) is similar to the estimate (∼20 h) in Moskvina and Schmidt [6].

**Table 1:** Running time for correcting genome-wide p-values in a study with 500 K SNPs over 5,000 individuals.

Procedure	# Permutations	Permutation	SLIDE	DSA	Mvtnorm*	RAT	Keffective
Correcting 1 p-value	10 K	16 d	0.6 h	1.4 h	0.7 h	7 h	19 h
Correcting 10 p-values	10 K	16 d	0.6 h	14 h	7 h	70 h	19 h
Correcting 1 p-value	100 K	160 d	6 h	14 h	7 h	72 h	19 h
Correcting 10 p-values	100 K	160 d	6 h	140 h	70 h	30 d	19 h
Correcting 1 p-value	1 M	4 years	3 d	6 d	3 d	30 d	19 h
Correcting 10 p-values	1 M	4 years	3 d	60 d	30 d	300 d	19 h

*: Often anti-conservative.

All values are extrapolated from the chromosome 22 results.

**Figure 6:**
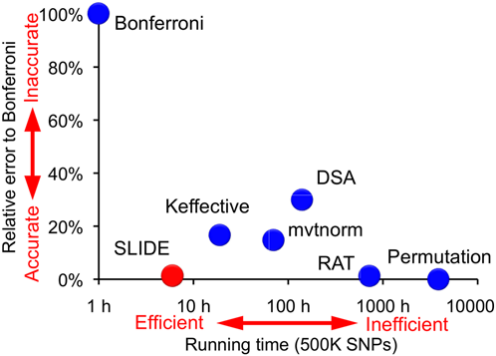
SLIDE's accuracy and efficiency compared to other methods. We use the WTCCC T2D case/control chromosome 22 data. The vertical axis is the average error in corrected p-values relative to the Bonferroni correction. The horizontal axis is the approximated time for correcting 10 genome-wide p-values for 500 K SNPs assuming 100 K permutations.

In many settings, SLIDE is 500 times faster than the permutation test and considerably faster than the other methods. The running time of SLIDE, Keffective, DSA, and mvtnorm is approximately independent of the study sample size, whereas the time of the permutation test is linearly dependent on it. Thus, the efficiency gain of these methods relative to the permutation test will increase as the study size increases. We summarize the accuracy and efficiency of the tested methods in Figure 6.

Here we describe a few details of our running time measurements. We used our own C implementation for the permutation test. However, we expect that the measured time will be similar to that for commonly used software such as PLINK [8], based on the claimed running time of PLINK on its website (1 CPU-day for 50 k permutations over 100 K SNPs of 350 samples). Note that PLINK's default “adaptive permutation” is a single SNP permutation to estimate the pointwise p-value, thus its max(T) permutation is required for multiple testing correction. Measuring the running time of mvtnorm has some subtleties since it has two parameters, the number of samples (maxpts) and the absolute error (abseps). The procedure is terminated if either the maximum number of samples is reached or the specified error is obtained. Therefore, we set abseps to a very small level (10^−20^) so that the specified number of samples will always be sampled. RAT also has some subtleties involving accuracy and efficiency. If we drop the -X -e2 parameters for an approximated importance sampling, RAT becomes much faster, but the resulting p-values are underestimated by a factor of up to 5 (data not shown). We assumed a corrected p-value of 10^−4^ to calculate the number of iterations for RAT using the formula presented in Kimmel and Shamir [30]. Since the formula is conservative, the running time of RAT may be overestimated. The constant window size of 100 may be too large for Keffective, since its purpose is to efficiently approximate the estimate. With a window size of 10, Keffective takes only 2 hours for 500 K SNPs. However, if we reduce the window size, the time for other methods including SLIDE will also be reduced.

Using the same WTCCC chromosome 22 dataset, we perform an additional experiment for the unphased genotype data using the trend test, assuming unbalanced case/controls. We find SLIDE achieves similar accuracy (See Text S4 and Figure S2).

#### Per-marker threshold estimation using all SNPs in HapMap

In this experiment, we assume that a single threshold is being estimated to decide which findings to follow up, instead of correcting each pointwise p-value. We estimate the per-marker threshold corresponding to a significance threshold of .05. We use the 2.7 million polymorphic SNPs in the HapMap CEU data over the whole genome, instead of a single chromosome.

We generate a simulated dataset using the phased haplotype data of 60 HapMap CEU parental individuals. Specifically, we create a new haplotype by randomly shuffling the 120 chromosomes so that the average length of a haplotype segment is approximately 1 Mb. We mutate (flip) each SNP with probability 10^−5^. We create 2,000 cases and 2,000 controls by randomly pairing 8,000 such haplotypes. Although this model is arbitrary, it suffices to compare different methods. The results of the relative comparison between methods do not greatly vary using different parameters, such as a different average haplotype segment length (data not shown).

We compare the permutation test, Keffective, and SLIDE. RAT is not efficient for this setting because it is optimized for very significant p-values, much smaller than .05. We expect that the results of DSA or mvtnorm will be similar to or worse than those of Keffective, as in the previous experiment.

**Figure 7:**
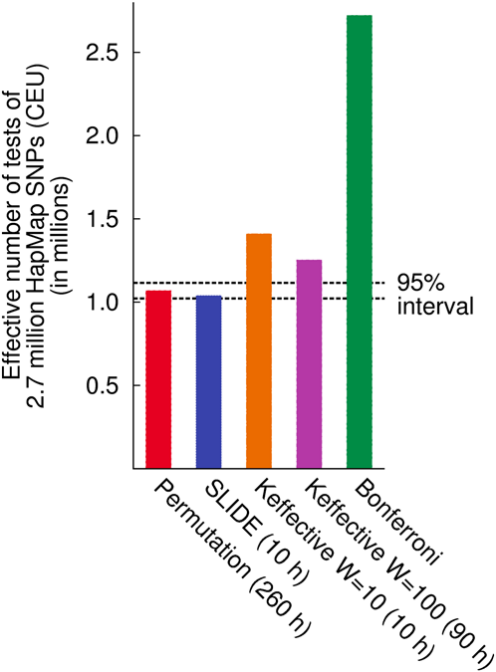
Effective number of tests of the 2.7 million HapMap SNPs for a simulated dataset. A dataset of 2,000 cases and 2,000 controls is generated from the HapMap CEU data. Using each method, we estimate the per-marker threshold corresponding to a significance level of .05. The effective number of test is simply .05 divided by the per-marker threshold. The dashed lines denote the interval where an accurate methods' estimate will be found more than 95% of the time.

We perform 10 K permutations for this experiment. We run SLIDE with 10 K samplings and window size 100. We run Keffective with window sizes 100 and 10. Figure 7 shows the “effective number of tests” estimated by each method, which is simply the significance threshold (.05) divided by the estimated per-marker threshold. The permutation test estimates the effective number of tests as 1,068,746 out of 2,721,223 tests. Thus, the Bonferroni correction is conservative by 155%. Note that in the previous experiment with a less-dense SNP set, the Bonferroni correction was conservative by 64%. The Bonferroni correction's error will continue to increase with the marker density.

The dashed lines denote the interval where an accurate methods' estimate will be found more than 95% of the time. SLIDE estimates the effective number as 1,038,888 (2.8% error), which is within the 95% interval. This small anti-conservative error is only due to the stochastic error and not an inherent bias, since the result becomes highly accurate as 1,068,445 (0.03% error) if we increase the number of samples to 100 K.

Keffective estimates the effective number as 1,409,811 (32% error) with window size 10 and as 1,252,986 (17% error) with window size 100. Unlike the previous experiment, for this higher-density marker dataset, Keffective no longer keeps the error within 10%. We do not expect that a larger window size will increase the accuracy of Keffective, because the error does not seem to be due to the missing long range correlations, since SLIDE is accurate with the same window size of 100.

The running time is 260 hours for permutation, 10 hours for SLIDE, 10 hours for Keffective with window size 10, and 90 hours for Keffective with window size 100.

#### Window size

Since SLIDE takes into account only correlations within the window size, here we investigate the effect of window size on performance. A reasonable choice for the window size will be the number of markers whose average distance is the average or maximum LD distance in the data. For our experiments, we use the WTCCC T2D case/control chromosome 22 dataset. A large number (10 M) of permutations allows us to find that a pointwise p-value 1.53×10^−5^ corresponds to the corrected p-value .05. We correct this pointwise p-value using SLIDE with various window sizes, and see if the corrected p-values are close to .05.

**Figure 8:**
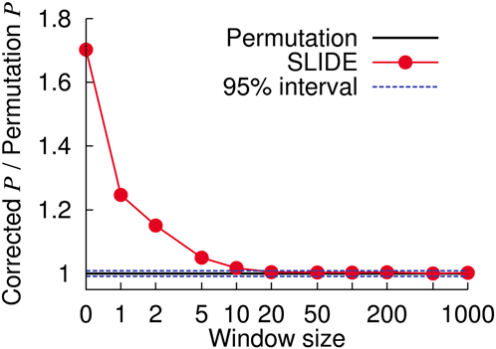
Effect of window size on SLIDE's performance. Using the WTCCC T2D case/control chromosome 22 data, we plot the ratios between the corrected p-value and the permutation p-value for varying window sizes for SLIDE. We use the pointwise p-value corresponding to the permutation p-value .05. The window size zero denotes the Bonferroni correction. The dashed lines denote the interval where an accurate methods' estimate will be found more than 95% of the time.

Figure 8 shows the ratio between the corrected p-value and the permutation p-value (.05) for various window sizes. Window size zero denotes the Bonferroni correction. The estimate is within the 95% interval for window sizes greater than 20, showing that this is the minimum choice of the window size for this dataset. In this dataset, the average distance between 20, 50, and 100 markers are approximately 100 Kb, 300 Kb, and 600 Kb.

#### Multiple testing correction using reference dataset

We now examine whether the per-marker threshold estimated from the reference dataset can approximate the true per-marker threshold for a study which may have a different sample correlation structure from the reference dataset. The marker set we use is the SNPs in the Affymetrix 500 K chip over the whole genome.

First, we apply SLIDE to the HapMap data using window size 100, to obtain the per-marker threshold 2.19×10^−7^ corresponding to the significance threshold .05. Then, we permute the WTCCC data to estimate the false positive rate given this per-marker threshold. We use the WTCCC 1958 British birth cohort control data, which consists of 1,504 individuals. We randomly permute the dataset 100 K times. We estimate the false positive rate, as the proportion of permutations showing significance given the per-marker threshold, to be .0508. Thus, in this experiment, the per-marker threshold estimated from the reference data controls the false positive rate with only 1.6% relative error. This result shows that, even if the reference population and the target population are slightly different (one from the Utah, U.S.A., and the other from the Great Britain), the per-marker threshold estimated from the reference data is a reasonable approximation.

### Power Estimation

We compare four different methods for estimating genome-wide power: standard simulation, null/alternative panel construction, best-tag Bonferroni, and SLIP. We assume a multiplicative disease model with a relative risk of 1.2 and a disease prevalence of .01, and a significance threshold of .05. We use the CEU population data in the HapMap as the reference dataset. We use the genome-wide markers in the Affymetrix 500 K chip and assume a uniform distribution of causal SNPs over all common SNPs (MAF≥.05) in the HapMap.

We first perform the standard simulation, which we will consider as the gold standard. We construct a number of genome-wide ‘alternative’ panels from the HapMap data by randomly assigning a causal SNP for each panel. We permute each panel 1,000 times to estimate the panel-specific per-marker threshold. The power is estimated as the proportion of panels showing significance given its per-marker threshold. Conneely and Boehnke [4] used this procedure for power estimation.

Another panel construction-based approach is the null/alternative panel construction method. Instead of permuting each of alternative panels, this method constructs another set of ‘null’ panels under the null hypothesis. The null panel gives us a ‘global’ per-marker threshold that can be applied to all alternative panels. Since this method is as accurate as the standard simulation but is more efficient, it is widely used [17],[18],[21].

We apply SLIP and re-use the samples for the null MVN for estimating the alternative MVNs. Lastly, we apply the analytical best-tag Bonferroni method [19]–[21] which uses the Bonferroni correction for the per-marker threshold and estimates power for each causal SNP by using the most correlated marker (best tag SNP). This method can also be accelerated by sampling the causal SNPs and setting a window size.

For the standard simulation, we use 10 K alternative panels. For the null/alternative panel construction method, we use 10 K alternative panels and 10 k null panels. For SLIP, we use 10 K sampling points. For the best-tag Bonferroni method, we use 10 K samples for causal SNPs. For SLIP, we use a window size of 100 markers. For all other methods, we use a window size of 1 Mb.

**Figure 9:**
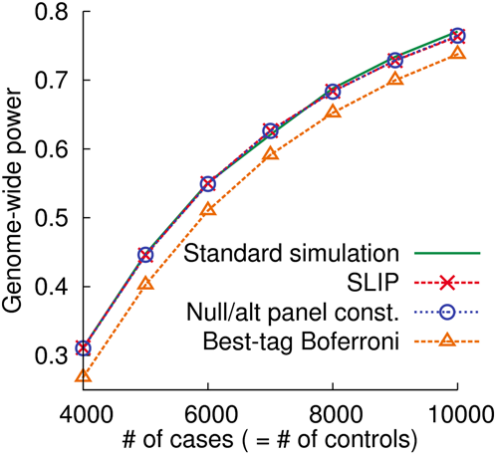
Genome-wide power of the Affymetrix 500 k chip estimated by different methods. We use the HapMap CEU reference data. We assume a multiplicative disease model with relative risk 1.2, disease prevalence .01, and a uniform distribution of causal SNPs over common SNPs (MAF≥.05). We use the significance threshold of .05.

Figure 9 shows that both SLIP and the null/alternative panel construction method are as accurate as the standard simulation. The best-tag Bonferroni method is inaccurate, underestimating power by up to 5%.

Table 2 shows the running time of each method for estimating genome-wide power. As shown, SLIP is very efficient. Since SLIP uses the correlation structure, the running time is approximately independent of the study sample size, whereas the running time of the standard simulation or the null/alternative panel construction method is linearly dependent on the sample size.

**Table 2:** Running time for estimating genome-wide power with 10 K samplings.

Procedure	#cases/controls	Best-tag-Bonf.*	SLIP	Null/altern.	Std. simul.
Estimating power	1,000/1,000	0.1 h	0.6 h	36 h	10 d
	5,000/5,000	0.1 h	0.6 h	8 d	50 d
Estimating power for 5 different relative risks	1,000/1,000	0.1 h	0.6 h	8 d	50 d
	5,000/5,000	0.1 h	0.6 h	40 d	250 d

*: Inaccurate (average error is not within 1%).

## Discussion

SLIDE and SLIP provide efficient and accurate multiple testing correction and power estimation in the MVN framework. SLIDE shows a near identical accuracy to the permutation test by using a sliding-window approach to account for local correlations, and by correcting for the error caused by using the asymptotic approximation. SLIDE can be applied to datasets of millions of markers with many rare SNPs, while other MVN-based methods become inaccurate as more rare SNPs are included. To the best of our knowledge, SLIP is the first MVN-based power estimation method.

Throughout this paper, we considered the classical multiple testing correction controlling family-wise error rate (FWER) [7], the probability of observing one or more false positives. SLIDE can be extended to control false discovery rate [41],[42] as well, using a similar approach to Lin [10]. In Text S1, we show that the MVN framework can be extended to the weighted haplotype test [15],[16] and the test for imputed genotypes [17]. SLIDE can be use for any multiple testing correction problem with a local correlation structure, as long as the covariance between statistics can be derived.

We considered the permutation test as the gold standard for multiple testing correction. The permutation test can be performed in two different ways: at each permutation, we can either assess the maximum statistics among the markers (max-T permutation), or assess the minimum pointwise p-value among the markers by performing another permutation for each marker (min-P permutation) [7],[42]. We used the former approach because the latter approach is computationally very intensive.

In Text S5 and Figure S3, we describe some additional insights obtained through the study. When marker frequencies do not follow the Hardy-Weinberg proportions (HWP), the use of an allelic test (e.g. allelic 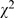 test) for unphased genotype data is not recommended due to the possible bias [43]. However, widely used software [8] often allows the use of an allelic test for genotype data under the reasoning that, as long as the permutation or an exact test is performed, the pointwise p-value will be the same as if we use a genotypic test (e.g. Armitage's trend test). Theoretically, this is due to the fact that the allelic and genotypic test statistics differ only by their variance [44]. However, for assessing corrected p-values, the permutation test does not provide this kind of “protection”. Even after a quality control process that excludes SNPs which significantly deviate from the Hardy-Weinberg equilibrium (HWE), still many SNPs may not follow HWP. Therefore, using an allelic test for genotype data for multiple testing correction can result in inaccurate estimates.

Recently, a different view of multiple testing correction has been introduced [5],[28], which suggest that we should correct for the uncollected or unknown markers as well as the collected markers, in order to take into account additional testing burdens such as the possible testings in a follow-up study. Pe'er *et al.* [28] estimates the per-marker threshold by extrapolating from the resequenced ENCODE regions, and Dudbridge *et al.* [5] estimates the per-marker threshold by subsampling the SNPs at an increasing SNP density. Although we employed the classical point of view that corrects for multiple testing only over observed SNPs, our method can also be applied to this alternative view. Our method can be used to estimate the effective number of tests for a representative resequenced region or for the set of subsampled SNPs. Since the SNP density of genotyping technology is dramatically increasing, we assume that the number of unknown and uncollected SNPs will decrease, causing the two different views to converge.

In our experiments, we used a constant block size for the block-wise strategy. In practice, it will be more reasonable to split the region according to the LD blocks. However, this is not always possible because LD blocks are often ambiguous and some blocks can be larger than the maximum block size of the method. For example, if we collect 10 million SNPs, a block size of 1,000 is required to cover 300 kb LD. However, the maximum block size of mvtnorm that allows an accurate estimate is currently 300 [4], and DSA with window size 1,000 often requires a prohibitively large memory in our simulations (data not shown). By contrast, SLIDE with window size 1,000 for the WTCCC chromosome 22 data requires ∼150 Mb memory and thus is feasible. Nevertheless, it should be noted that the block-wise strategy can always be implemented to have the same block size as SLIDE.

Recently, a method called PRESTO [45] was introduced, which increases the efficiency of the permutation test by applying several optimization techniques. Based on the claimed running time, SLIDE is ∼10 times faster than PRESTO, but PRESTO has an advantage that it does not depend on the asymptotic approximation but provides exactly the same result as the permutation test.

We considered the pairwise correlation between SNPs. There can also be so-called higher-order correlations, such as the correlation between a haplotype and a SNP. For example, even though three SNPs are pairwisely independent, the combination of the first two SNPs can be a perfect proxy to the third SNP. However, the multivariate central limit theorem proves that the joint distribution of the test statistics is fully characterized by the matrix of the pairwise correlations. Thus, the effect of the other correlation terms on the joint distribution is asymptotically negligible. Nevertheless, our method is not limited to the SNP test. If our method is applied to the weighted haplotype test [15],[16] as shown in Text S1, the pairwise correlation in the correlation matrix can be interpreted as the higher-order correlations between a haplotype and a SNP or between haplotypes.

In summary, SLIP and SLIDE are two useful methods for genome-wide association studies which provide accurate power estimation at the design step and accurate multiple testing correction at the analysis step. The software is available as a resource for the research community.

## Supporting Information

Figure S1 Ratios between the corrected p-values and permutation p-values after rare SNPs are removed. We use the chromosome 22 of the WTCCC Type 2 diabetes cases/controls data. Multiple testing is corrected with respect to the 4,515 common SNPs (MAF≥.05).
(0.01 MB PDF)

Figure S2 Ratios between the corrected p-values and permutation p-values for genotype data. We simulate a unphased genotype dataset using the chromosome 22 data of the WTCCC Type 2 diabetes cases/controls data, assuming a unbalanced study of 2,934 controls and 1,928 cases.
(0.01 MB PDF)

Figure S3 Inaccurate multiple testing correction caused by the use of an allelic test for unphased genotype data. We generate a simulated unphased genotype data of 120 cases and 120 controls from the HapMap CEU population chromosome 22 data. Then we plot the ratios between the corrected p-values by two different permutations: permutation test using the allelic test statistic, and permutation test using the genotypic test statistic. Quality control is performed by the standard χ^2^ test for HWE.
(0.01 MB PDF)

Text S1 Rapid and accurate multiple testing correction and power estimation for millions of correlated markers.
(0.14 MB PDF)
