# Supplementary figures and images for: Rapid and Accurate Multiple Testing Correction and Power Estimation for Millions of Correlated Markers
Source: PLoS Genet. 2009 Apr 17;5(4):e1000456. doi: 10.1371/journal.pgen.1000456 (PMC2663787; doi:10.1371/journal.pgen.1000456)

# Rare SNP-eliminated analysis

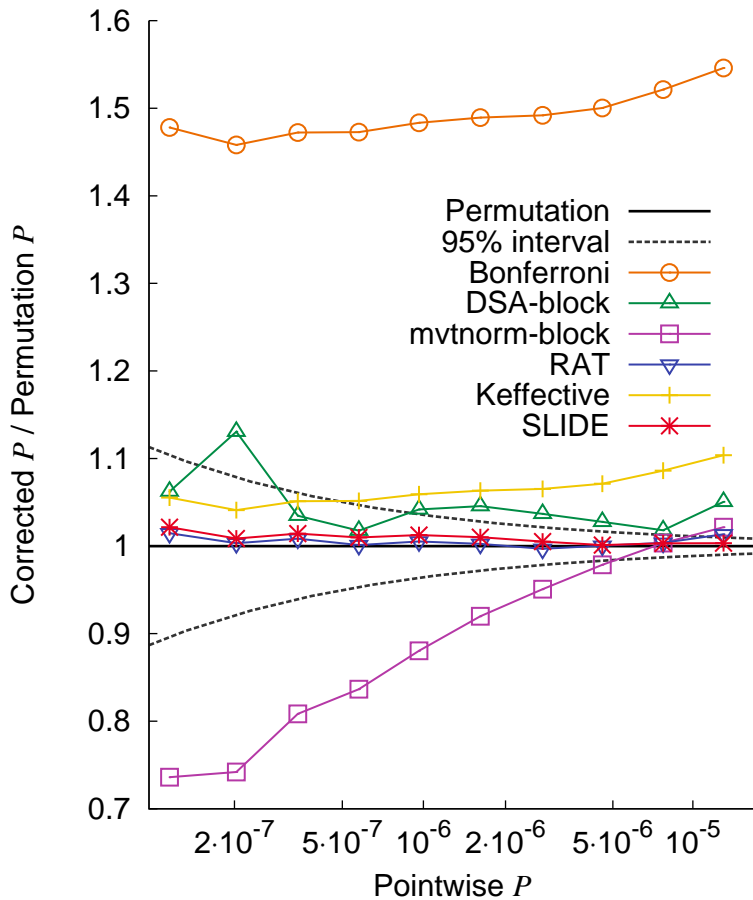

Supplement: Figure S1 — Ratios between the corrected p-values and permutation p-values after rare SNPs are removed. We use the chromosome 22 of the WTCCC Type 2 diabetes cases/controls data. Multiple testing is corrected with respect to the 4,515 common SNPs (MAF≥.05). (0.01 MB PDF) [file pgen.1000456.s001.pdf]

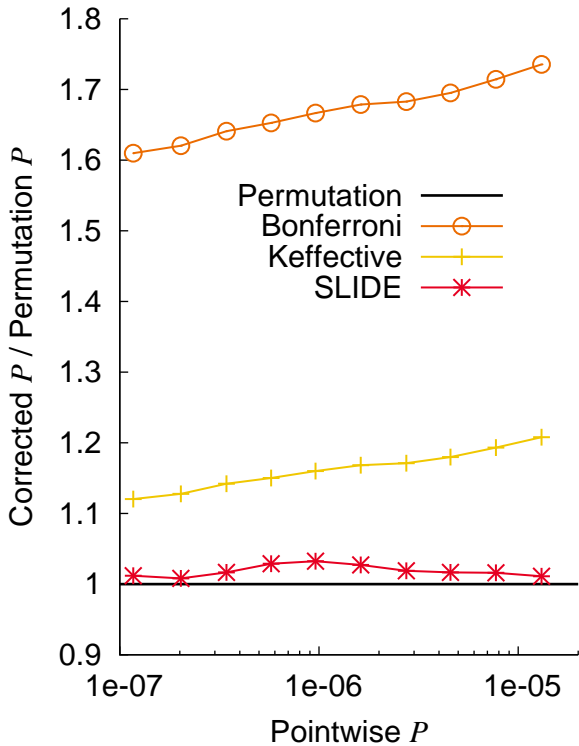

Supplement: Figure S2 — Ratios between the corrected p-values and permutation p-values for genotype data. We simulate a unphased genotype dataset using the chromosome 22 data of the WTCCC Type 2 diabetes cases/controls data, assuming a unbalanced study of 2,934 controls and 1,928 cases. (0.01 MB PDF) [file pgen.1000456.s002.pdf]

Corrected  $P$  by each method /  
Gold standard corrected  $P$

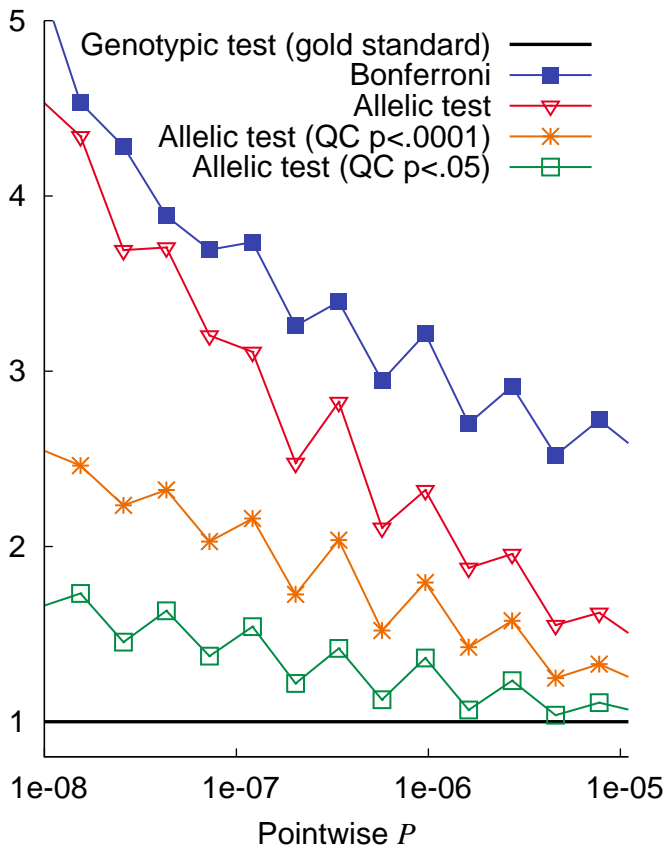

Supplement: Figure S3 — Inaccurate multiple testing correction caused by the use of an allelic test for unphased genotype data. We generate a simulated unphased genotype data of 120 cases and 120 controls from the HapMap CEU population chromosome 22 data. Then we plot the ratios between the corrected p-values by two different permutations: permutation test using the allelic test statistic, and permutation test using the genotypic test statistic. Quality control is performed by the standard χ2 test for HWE. (0.01 MB PDF) [file pgen.1000456.s003.pdf]
