# Supplementary material for: Rapid and Accurate Multiple Testing Correction and Power Estimation for Millions of Correlated Markers
Source: PLoS Genet. 2009 Apr 17;5(4):e1000456. doi: 10.1371/journal.pgen.1000456 (PMC2663787; doi:10.1371/journal.pgen.1000456)
Supplement: Text S1 — Rapid and accurate multiple testing correction and power estimation for millions of correlated markers. (0.14 MB PDF) [file pgen.1000456.s004.pdf]

Supplementary Text: Rapid and Accurate Multiple Testing Correction and Power Estimation for Millions of Correlated Markers

Buhm Han[1], Hyun Min Kang[1], Eleazar Eskin[2,*]

1 Department of Computer Science and Engineering, University of California, San Diego, La Jolla, California, United States of America

2 Department of Computer Science and Department of Human Genetics, University of California, Los Angeles, Los Angeles, California, United States of America

*** E-mail: eeskin@cs.ucla.edu**

S1 Covariance of various test statistics

Weighted haplotype test

Suppose we randomly permute the dataset and apply the weighted haplotype test [1, 2], which can be considered a type of imputation statistic. Assume we have $N/2$ cases and $N/2$ controls (total $2N$ chromosomes). Consider a ungenotyped marker i. By using nearby genotyped markers, we define k haplotypes. Let p_h be the frequency of the haplotype h in the overall sample, which is constant regardless of the permutation. Let $\hat{p}_h^+$ and $\hat{p}_h^-$ be the observed frequency of the haplotype h in the permuted cases and controls respectively. Let q_{ih} be the conditional probability observing the minor allele at marker i given the haplotype h. q_{ih} can be estimated from the reference dataset, and is invariant between cases and controls under the null. Then, the test statistic at ungenotyped marker i is

$$S_i = \sqrt{\frac{2N-1}{4}} \frac{\sum_{h=1}^{k} q_{ih}(\hat{p}_h^+ - \hat{p}_h^-)}{\sqrt{\sum_h q_{ih}^2 p_h - (\sum_h q_{ih} p_h)^2}} \sim \mathcal{N}(0,1) \ .$$

S_i differs from the statistic of Zaitlen *et al.* [1] or Nicolae [2] by a factor of $\sqrt{\frac{2N-1}{2N}}$ due to the without-replacement sampling of the permutation test.

Now we consider the covariance between statistics S_i at ungenotyped marker i and S_j at ungenotyped marker j. The covariance between a genotyped marker and a ungenotyped marker can be straightforwardly derived, because a genotyped marker can always be considered as a ungenotyped marker with a nearby haplotype of size 1 which is the marker itself. Let h be the one of k nearby haplotypes defined for marker i and h' be the one of k' nearby haplotypes defined for marker j. Let $p_{hh'}$ be the frequency of the overall sample chromosomes which have both haplotype h and haplotype h'. Let $\hat{p}_{hh'}^+$ and $\hat{p}_{hh'}^-$ be the observed frequencies in the cases and controls after random permutation. Similarly to the derivation

in the main text,

$$\begin{aligned}
\text{Cov}\left(\hat{p}_h^+, \hat{p}_{h'}^+\right) &= \frac{1}{2N-1}\left(p_{hh'} - p_h p_{h'}\right) \\
\text{Cov}\left(\hat{p}_h^+ - \hat{p}_h^-, \hat{p}_{h'}^+ - \hat{p}_{h'}^-\right) &= \text{Cov}\left(\hat{p}_h^+ - (2p_h - \hat{p}_h^+), \hat{p}_{h'}^+ - (2p_{h'} - \hat{p}_{h'}^+)\right) \\
&= \text{Cov}\left(2\hat{p}_h^+ - 2p_h, 2\hat{p}_{h'}^+ - 2p_{h'}\right) \\
&= \text{Cov}\left(2\hat{p}_h^+, 2\hat{p}_{h'}^+\right) \\
&= 4\text{Cov}\left(\hat{p}_h^+, \hat{p}_{h'}^+\right) \\
&= \frac{4}{2N-1}\left(p_{hh'} - p_h p_{h'}\right)
\end{aligned}$$

Thus,

$$\text{Cov}\left(S_i, S_j\right) = \frac{\sum_{h=1}^{k}\sum_{h'=1}^{k'} q_{ih} q_{jh'}\left(p_{hh'} - p_h p_{h'}\right)}{\sqrt{\left(\sum_h q_{ih}^2 p_h - (\sum_h q_{ih} p_h)^2\right)\left(\sum_{h'} q_{jh'}^2 p_{h'} - (\sum_{h'} q_{jh'} p_{h'})^2\right)}}$$

Test for imputed genotypes based on posterior probabilities

A different type of imputation statistic is the test for imputed genotypes based on the posterior probabilities. Since many imputation softwares [3] provide the posterior probabilities for genotypes, we will consider the genotypic test instead of the allelic test. We derive the covariance by using the score statistic.

Unambiguous genotypes If the genotypes are unambiguous, the standard score test can be used as in Seaman and Müller-Myhsok [4]. Assume $N/2$ cases and $N/2$ controls. Let Y_i be the binary trait of individual i. Let $Z_i \in \{0,1,2\}^M$ be the vector of M marker genotypes of individual i. Let α be the intercept term, and let β be the vector of coefficients describing the effect of the genotypes on the trait. Under the logistic regression model such that

$$P(Y_i = 1|Z_i, \alpha, \beta) = \tilde{Y}_i = \frac{e^{\alpha+\beta Z_i}}{1+e^{\alpha+\beta Z_i}} \ ,$$

the likelihood of $\theta = (\alpha, \beta)$ is

$$L(\theta) = \prod_{i=1}^{N} \exp\Big(Y_i \eta_i - \ln\big(1 + \exp(\eta_i)\big)\Big) \ ,$$

where $\eta_i = \alpha + \beta Z_i$.

The score and the information matrix are

$$\begin{aligned}
U(\theta) &= \sum_{i=1}^{N}(Y_i - \tilde{Y}_i)(1 \ Z_i^T)^T \\
I(\theta) &= \sum_{i=1}^{N}\tilde{Y}_i(1-\tilde{Y}_i)(1 \ Z_i^T)^T(1 \ Z_i^T) \ .
\end{aligned}$$

Let $\hat{\alpha}_0$ be the maximum likelihood estimate of α under the null hypothesis of $\beta = \beta_0 = 0$, which is zero for this balanced case/control study. The score and the information matrix for the genetic markers are obtained by correcting for the intercept term and apply $\theta_0 = (\hat{\alpha}_0, \beta_0) = (0, 0)$,

$$\begin{aligned} U_\beta &= U(\theta_0)_\beta \\ I_\beta &= I(\theta_0)_{\beta\beta} - I(\theta_0)_{\beta\alpha} I(\theta_0)^{-1}_{\alpha\alpha} I(\theta_0)_{\alpha\beta} \; . \end{aligned}$$

The score test statistic $U_\beta^T I_\beta^{-1} U_\beta$ asymptotically follows a $\chi^2_{\mathrm{df}=M}$ distribution. The test statistic at marker j, $S_j = \frac{U_{\beta(j)}}{\sqrt{I_{\beta(j,j)}}} \sim \mathcal{N}(0,1)$ is equivalent to the Cochran-Armitage trend test statistic. The covariance between two statistics S_j and S_k is $\frac{I_{\beta(j,k)}}{\sqrt{I_{\beta(j,j)} I_{\beta(k,k)}}}$, which is the Pearson correlation coefficient r.

Ambiguous genotypes (imputation) If the genotypes are ambiguous and only the posterior probabilities are given, we should take into account the uncertainty in the score statistic as in Marchini *et al.* [3] and Schaid *et al.* [5]. Let $\mathbb{E}_p[\cdot]$ be the expectation over the posterior distribution.

As described in Louis [6], the score and the information matrix with missing data are

$$\begin{aligned} U^*(\theta) &= \sum_{i=1}^{N} (Y_i - \tilde{Y}_i)(1 \; \mathbb{E}_p[Z_i^T])^T \\ I^*(\theta) &= \sum_{i=1}^{N} \mathbb{E}_p[I] - \sum_{i=1}^{N} \Big(\mathbb{E}_p[UU^T] - \mathbb{E}_p[U]\mathbb{E}_p[U^T] \Big) \end{aligned}$$

where U and I are the score and information when we assume the data are complete. The first term, $\mathbb{E}_p[I]$, is the variance of assuming a complete dataset, and the second term is the penalty of having uncertainty in the data.

$$\begin{aligned} I^*(\theta) &= \sum_{i=1}^{N} \tilde{Y}_i (1 - \tilde{Y}_i) \mathbb{E}_p\left[(1 \; Z_i^T)^T (1 \; Z_i^T) \right] \\ &\quad - \sum_{i=1}^{N} (Y_i - \tilde{Y}_i)^2 \Big(\mathbb{E}_p\left[(1 \; Z_i^T)^T (1 \; Z_i^T) \right] - \mathbb{E}_p\left[(1 \; Z_i^T)^T \right] \mathbb{E}_p\left[(1 \; Z_i^T) \right] \Big) \\ I^*(\theta)_{\alpha\alpha} &= \sum_{i=1}^{N} \tilde{Y}_i (1 - \tilde{Y}_i) \\ I^*(\theta)_{\alpha\beta} &= \sum_{i=1}^{N} \tilde{Y}_i (1 - \tilde{Y}_i) \mathbb{E}_p[Z_i] \\ I^*(\theta)_{\beta\beta} &= \sum_{i=1}^{N} \tilde{Y}_i (1 - \tilde{Y}_i) \mathbb{E}_p\left[Z_i Z_i^T \right] - \sum_{i=1}^{N} (Y_i - \tilde{Y}_i)^2 \left(\mathbb{E}_p\left[Z_i Z_i^T \right] - \mathbb{E}_p\left[Z_i \right] \mathbb{E}_p\left[Z_i^T \right] \right) \end{aligned}$$

Under the null, for this balanced case/control study, $\hat{\alpha}_0 = 0$ and $\tilde{Y}_i = \frac{1}{2}$. Thus,

$$
\begin{aligned}
I^*(\theta_0)_{\alpha\alpha} &= \frac{1}{2} \cdot \frac{1}{2} \cdot N = \frac{N}{4} \\
I^*(\theta_0)_{\alpha\beta} &= \frac{1}{4} \sum_{i=1}^{N} \mathbb{E}_p[Z_i] \\
I^*(\theta_0)_{\beta\beta} &= \frac{1}{4} \sum_{i=1}^{N} \mathbb{E}_p \left[Z_i Z_i^T \right] - \sum_{i=1}^{N} \left(Y_i - \frac{1}{2} \right)^2 \left(\mathbb{E}_p \left[Z_i Z_i^T \right] - \mathbb{E}_p \left[Z_i \right] \mathbb{E}_p \left[Z_i^T \right] \right) \\
U^*_\beta = U^*(\theta_0)_\beta &= \sum_{i=1}^{N} \left(Y_i - \frac{1}{2} \right) \mathbb{E}_p[Z_i] \\
I^*_\beta &= I^*(\theta_0)_{\beta\beta} - I^*(\theta_0)_{\beta\alpha} I^*(\theta_0)^{-1}_{\alpha\alpha} I^*(\theta_0)_{\alpha\beta} \\
&= \frac{1}{4} \left(\sum_{i=1}^{N} \mathbb{E}_p \left[Z_i Z_i^T \right] - \frac{\sum_{i=1}^{N} \mathbb{E}_p[Z_i] \sum_{i=1}^{N} \mathbb{E}_p[Z_i^T]}{N} \right) \\
&\quad - \sum_{i=1}^{N} \left(Y_i - \frac{1}{2} \right)^2 \left(\mathbb{E}_p \left[Z_i Z_i^T \right] - \mathbb{E}_p \left[Z_i \right] \mathbb{E}_p \left[Z_i^T \right] \right)
\end{aligned}
$$

The score test statistic is ${U^*}^T_\beta {I^*}^{-1}_\beta U^*_\beta$.

For the single marker test, if we denote the genotype at marker j of individual i as $Z_{i(j)}$, the statistic for marker j

$$
S_j = \frac{U^*_{\beta(j)}}{\sqrt{I^*_{\beta(j,j)}}} = \frac{\sum_{i=1}^{N} (Y_i - \frac{1}{2}) \mathbb{E}_p[Z_{i(j)}]}{\sqrt{\frac{1}{4} \left(\sum_{i=1}^{N} \mathbb{E}_p \left[Z^2_{i(j)} \right] - \frac{(\sum_{i=1}^{N} \mathbb{E}_p[Z_{i(j)}])^2}{N} \right) - \sum_{i=1}^{N} \left(Y_i - \frac{1}{2} \right)^2 \left(\mathbb{E}_p \left[Z^2_{i(j)} \right] - \mathbb{E}_p \left[Z_{i(j)} \right]^2 \right)}}
$$

follows the standard normal distribution. The covariance between S_j and S_k is given by $\frac{I^*_{\beta(j,k)}}{\sqrt{I^*_{\beta(j,j)} I^*_{\beta(k,k)}}}$, where

$$
\begin{aligned}
I^*_{\beta(j,k)} &= \frac{1}{4} \left(\sum_{i=1}^{N} \mathbb{E}_p \left[Z_{i(j)} Z_{i(k)} \right] - \frac{(\sum_{i=1}^{N} \mathbb{E}_p[Z_{i(j)}])(\sum_{i=1}^{N} \mathbb{E}_p[Z_{i(k)}])}{N} \right) \\
&\quad - \sum_{i=1}^{N} \left(Y_i - \frac{1}{2} \right)^2 \left(\mathbb{E}_p \left[Z_{i(j)} Z_{i(k)} \right] - \mathbb{E}_p \left[Z_{i(j)} \right] \mathbb{E}_p \left[Z_{i(k)} \right] \right) \\
I^*_{\beta(j,j)} &= \frac{1}{4} \left(\sum_{i=1}^{N} \mathbb{E}_p \left[Z^2_{i(j)} \right] - \frac{(\sum_{i=1}^{N} \mathbb{E}_p[Z_{i(j)}])^2}{N} \right) - \sum_{i=1}^{N} \left(Y_i - \frac{1}{2} \right)^2 \left(\mathbb{E}_p \left[Z^2_{i(j)} \right] - \mathbb{E}_p \left[Z_{i(j)} \right]^2 \right) \\
I^*_{\beta(k,k)} &= \frac{1}{4} \left(\sum_{i=1}^{N} \mathbb{E}_p \left[Z^2_{i(k)} \right] - \frac{(\sum_{i=1}^{N} \mathbb{E}_p[Z_{i(k)}])^2}{N} \right) - \sum_{i=1}^{N} \left(Y_i - \frac{1}{2} \right)^2 \left(\mathbb{E}_p \left[Z^2_{i(k)} \right] - \mathbb{E}_p \left[Z_{i(k)} \right]^2 \right) .
\end{aligned}
$$

For this balanced case/control study, since $\left(Y_i - \frac{1}{2}\right)^2 = \frac{1}{4}$, the formula simplifies to

$$\mathrm{Cov}\left(S_j, S_k\right) = \frac{\sum_{i=1}^{N} \mathbb{E}_p\left[Z_{i(j)}\right]\mathbb{E}_p\left[Z_{i(k)}\right] - (\sum_{i=1}^{N}\mathbb{E}_p[Z_{i(j)}])(\sum_{i=1}^{N}\mathbb{E}_p[Z_{i(k)}])/N}{\sqrt{\left(\sum_{i=1}^{N}\mathbb{E}_p\left[Z_{i(j)}\right]^2 - (\sum_{i=1}^{N}\mathbb{E}_p[Z_{i(j)}])^2/N\right)\left(\sum_{i=1}^{N}\mathbb{E}_p\left[Z_{i(k)}\right]^2 - (\sum_{i=1}^{N}\mathbb{E}_p[Z_{i(k)}])^2/N\right)}}$$

Thus, the covariance is computable from the posterior probabilities.

However, if the study is unbalanced, computing covariance requires the knowledge of the joint posterior probabilities between markers ($\mathbb{E}_p\left[Z_{i(j)}Z_{i(k)}\right]$). To the best of our knowledge, no current imputation software provides the joint distribution. We assume that an algorithm can be developed to provide the joint distribution for a given window size W, however this will be beyond the scope of this paper.

S2 Details of sliding-window procedure in SLIDE

SLIDE approximates the MVN by using a sliding-window Monte-Carlo approach. Given M markers, let $(S_1, \cdots, S_M)$ be the vector of statistics which asymptotically follows a M-dimensional MVN with zero mean and variance Σ. Let $f(S_1, S_2, \cdots, S_M)$ be the probability density function of the statistics. Under the local LD assumption, statistics at distant markers are uncorrelated. Thus, given a window size w, we can assume that S_i is conditionally independent of $S_1, S_2, \cdots, S_{i-w-1}$ given $S_{i-w}, S_{i-w+1}, \cdots, S_{i-1}$. Then by the chain rule,

$$f(S_1, S_2, \cdots, S_M) = f(S_1)f(S_2|S_1)f(S_3|S_1, S_2)\cdots f(S_{M-1}|S_{M-w-1}, \cdots, S_{M-2})f(S_M|S_{M-w}, \cdots, S_{M-1})\ .$$

Using this equation, the following procedure samples a single M-dimensional random sample vector, based on the standard formula for conditional mean and variance in the MVN.

1. Sample $\hat{S}_1$ from $N(0, 1)$
2. $i \leftarrow 1$
3. $s \leftarrow \max(i - w, 1)$, $\hat{S}_{si} \leftarrow (\hat{S}_s, \hat{S}_{s+1}, \cdots, \hat{S}_i)$.
4. Let $\Sigma_{si,si}$ be a sub-matrix of Σ from index s to index i in both rows and columns, and let $\mathbf{v}_{si,(i+1)}$ be the sub-column vector of Σ with row index s to i, and column index $i + 1$.
5. Let $\mu \leftarrow \mathbf{v}_{si,(i+1)}^T \Sigma_{si,si}^{-1} \hat{S}_{si}$, $\sigma^2 \leftarrow 1 - \mathbf{v}_{si,(i+1)}^T \Sigma_{si,si}^{-1} \mathbf{v}_{si,(i+1)}$, and sample $\hat{S}_{i+1}$ from $N(\mu, \sigma^2)$.
6. $i \leftarrow i + 1$ and repeat from step 3 while $i < M$

We repeat this procedure to draw R sample vectors. Let $\hat{S}_i^j$ be the sample statistic for marker i in the j'th sample vector. The set of statistics $\{\hat{S}_i^j\}$ approximates the MVN. At step 5, we can reduce the computational overhead of repeatedly drawing random sample vectors by storing $\mathbf{v}_{si,(i+)}^T V_{si,si}^{-1}$ and σ^2 for each marker.

S3 Conditional probabilities in cases and controls

Let c denote the causal SNP, A denote the marker, and $+$ denote the diseased status. We show that the conditional probability, $P_{A|c}$, does not depend on the disease status and thus is invariant among case, control, and overall populations.

$$
\begin{aligned}
P^{+}_{A|c} &= P(A|c,+) \\
&= \frac{P(A,c|+)}{P(c|+)} \\
& \text{By Bayes' rule,} \\
&= \frac{P(+|A,c)P(A,c)}{P(+|A,c)P(A,c)+P(+|A,C)P(A,C)+P(+|a,c)P(a,c)+P(+|a,C)P(a,C)} \cdot \frac{1}{P(c|+)} \\
& \text{Since } A \text{ does not affect the disease status given } c, \\
&= \frac{P(+|c)P(A,c)}{P(+|c)P(A,c)+P(+|C)P(A,C)+P(+|c)P(a,c)+P(+|C)P(a,C)} \cdot \frac{1}{P(c|+)} \\
&= \frac{P(+|c)P(A,c)}{P(+|c)P(c)+P(+|C)P(C)} \cdot \frac{1}{P(c|+)} \\
&= \frac{P(+|c)P(c)}{P(+|c)P(c)+P(+|C)P(C)} \cdot \frac{P(A,c)}{P(c)} \cdot \frac{1}{P(c|+)} \\
&= P(c|+) \cdot \frac{P(A,c)}{P(c)} \cdot \frac{1}{P(c|+)} \\
&= \frac{P(A,c)}{P(c)} \\
&= P_{A|c}
\end{aligned}
$$

The result goes along with our intuition; since the causal SNP is the only factor which causes the difference in haplotype distribution between cases and controls, once the causal SNP is conditioned, there is no more difference in haplotype distribution between cases and controls.

S4 P-value correction in Chromosome 22 of WTCCC data: genotype data and unbalanced study

In the main text of our article, we perform the p-value correction simulation using the chromosome 22 of the WTCCC dataset. We perform the same experiment, but instead of using the phased haplotype data assuming a balanced case/control study, we use the unphased genotype data assuming a unbalanced case/control study. We split the data into 2,934 controls and 1,928 cases. We correct ten different pointwise p-values using the permutation test, the Bonferroni correction, Keffective, and SLIDE. All other settings are the same as the simulation in the main text. Figure S2 shows that the average error rate of SLIDE is only 2%, while the error rate of the Bonferroni correction and Keffective are 67% and 16% respectively.

S5 Use of an allelic test for genotype data in multiple testing correction

Theory

We show that, given unphased genotype data, using an allelic test statistic can result in inaccurate multiple testing correction. Under the additive disease model, the natural choice of test statistic for unphased genotype data is the genotypic (Armitage's trend) test statistic. Allelic (χ^2) test can only be used if the SNP follows the exact expected frequencies of HWE (Hardy Weinberg proportions, or HWP). Unless, Sasieni [7] showed that the allelic test may be biased.

Specifically, if the number of genotypes in cases and controls at a SNP are given as follows,

# of minor allele	0	1	2	
Case	r_0	r_1	r_2	R
Control	s_0	s_1	s_2	S
	n_0	n_1	n_2	N

the allelic test statistic is

$$S_H^2 = \frac{2N\{2N(r_1+2r_2) - 2R(n_1+2n_2)\}^2}{(2R)(2S)\{2N(n_1+2n_2) - (n_1+2n_2)^2\}} ,$$

and the genotypic test statistic is

$$S_G^2 = \frac{N\{N(r_1+2r_2) - R(n_1+2n_2)\}^2}{RS\{N(n_1+4n_2) - (n_1+2n_2)^2\}} .$$

While the numerators of the two statistics are identical, the variances (denominators) differ. Devlin and Roeder [8] showed that

$$\rho^2 = \frac{S_H^2}{S_G^2} = 1 + \frac{4n_0n_2 - n_1^2}{(n_1+2n_2)(n_1+2n_0)} .$$

Thus, if the SNP does not follow HWP ($n_1^2 \neq 4n_0n_2$), the variance of S_G is 1 while the variance of S_H is ρ, which can be greater than or less than 1. This non-unit variance is caused by the fact that the cell count in the allelic 2×2 contingency table does not follow the hypergeometric distribution. For this reason, Sasieni [7] recommends against the use of allelic test for unphased genotype data.

For estimating pointwise p-value, however, widely used software such as PLINK [9] allows the use of allelic test for genotype data, because the use of permutation test or exact test provides a kind of protection for this deviation. Since the permutation test is only concerned about relative comparisons between statistics, as long as all statistics are scaled with a constant factor (ρ), the result is the same. Thus, the allelic test statistic may be safely used for unphased genotype data for estimating pointwise p-values.

Nevertheless, for estimating corrected p-values, the permutation test does not provide this kind of protection. When estimating pointwise p-value, we consider each SNP separately. For each single SNP, the ratio ρ is constant because n_0, n_1, and n_2 are fixed. Thus, we always have the relationship of

$S_H = \rho S_G$. That is, the relative order between the statistics obtained by multiple permutations is the same for the genotypic test and the allelic test. On the other hand, for estimating corrected p-values, we consider each SNP simultaneously. Permutation can be thought of as a procedure selecting the maximum statistic $\max(S_H)$ or $\max(S_G)$ among all markers at each permutation. Every SNP typically has a different ρ, because n_0, n_1, and n_2 vary between SNPs. Therefore, $\max(S_H)$ and $\max(S_G)$ do not have such a relationship as $S_H = \rho S_G$. That is, the relative order between the statistics obtained by multiple permutations may not be the same for the genotypic test and the allelic test. In the perspective of the MVN, while S_G follows the standard MVN whose variances are all 1 for every coordinate, S_H follows a "squeezed" MVN whose variances vary by each coordinate. Thus, the outside-rectangle probability of the MVN, which is considered an approximation of the corrected p-value, will be different from what we expect for this squeezed MVN. In other words, it may be difficult to accurately correct for multiple testing if we use the allelic test for unphased genotype data, even if we use the permutation test.

In fact, this error occurs because we are using the `max-T` style permutation which selects maximum statistic at each permutation. The goal is to identify the most significant result among all markers at each permutation. We can identify the most significant marker by performing `min-P` style permutation which involves performing an additional permutation to estimate each pointwise p-value. `Min-P` permutation is able to identify the most significant pointwise p-value even if the distribution of statistic is not identical among the markers. The use of an allelic test for genotype data makes the distribution of statistic not identical between markers by changing the variance of the normal distribution. Thus, the maximum statistic (under `max-T`) sometimes does not coincide with the most significant pointwise p-value. Nevertheless, the use of `min-P` permutation requires much more computational time than `max-T` permutation, which is impractical for large datasets.

Simulation

We first use the unphased genotype data of the WTCCC T2D case/control chromosome 22 dataset ($\sim$ 5K SNPs over 4,862 individuals). We perform the permutation test using two different test statistics: allelic test statistic and genotypic test statistic. We do not observe a large difference in corrected p-values between the two approaches. This is possibly because the deviation from the HWE is not very large, with an average $|F_{ST}|$ of .012.

We then construct a smaller dataset. We use 5,261 SNPs in the chromosome 22, but instead of using the WTCCC data, we construct a simulated dataset from the 60 parental individuals in the HapMap CEU population data. We copy the genotypes of individuals two times each to get 120 cases and 120 controls. This duplication procedure simulates Hardy-Weinberg disequilibrium by violating the random mating assumption. The average $|F_{ST}|$ of this dataset is as high as .103.

Then we simulate two different levels of quality control (QC). We perform χ^2 test for HWE for each SNP. In one experiment, we exclude 272 SNPs with HWE $p < .0001$ leaving 4,989 SNPs. In another experiment, we exclude 1,479 SNPs with HWE $p < .05$ leaving 3,782 SNPs. The average $|F_{ST}|$ of the two datasets are still high as .090 and .059. This is because passing the HWE test does not always mean that the SNP follows HWP.

Then we perform the permutation test using the genotypic test and the permutation test using the allelic test. We consider the p-values obtained by the permutation test using the genotypic test as the gold standard and plot the ratio between the gold standard and the p-values obtained by the permutation test using the allelic test, for each dataset of no QC, QC $p<.0001$, and QC $p<.05$. Figure S3 shows that using the allelic test for the unphased genotype data can result in inaccurate corrected p-values even closer to the Bonferroni correction than to the gold standard. After QC, the error is reduced, but a considerable amount of error still remains.

In this experiment, the use of allelic test results in conservative estimates. This can happen if, for example, the genotype calling error rate is much higher for the heterozygous allele than for the homozygous allele. However, the direction of the error can also be anti-conservative.

Although our simulation is unrealistic, it shows that the use of allelic test for unphased genotype data can result in inaccurate multiple testing correction, even after QC excluding SNPs which do not follow HWE. Our experiment using the WTCCC data shows that a large study with thousands of individuals is not much affected by this error, possibly because SNPs approximately follow HWP, but a study with hundreds of subjects can be affected. A simple solution to avoid this error is, given unphased genotype data, to use a genotypic test to obtain both pointwise p-values and corrected p-values and an allelic test to obtain pointwise p-values but not the corrected p-values until the data is phased. Another solution is to perform a `min-P` style permutation, but this will take a large amount of computational time.

References

1. Zaitlen N, Kang H, Eskin E, Halperin E (2007) Leveraging the HapMap correlation structure in association studies. Am J Hum Genet 80: 683–91.

2. Nicolae DL (2006) Testing untyped alleles (TUNA)-applications to genome-wide association studies. Genet Epidemiol 30: 718–727.

3. Marchini J, Howie B, Myers S, McVean G, Donnelly P (2007) A new multipoint method for genome-wide association studies by imputation of genotypes. Nature Genetics 39: 906-913.

4. Seaman SR, MÃŒller-Myhsok B (2005) Rapid simulation of P values for product methods and multiple-testing adjustment in association studies. Am J Hum Genet 76: 399–408.

5. Schaid DJ, Rowland CM, Tines DE, Jacobson RM, Poland GA (2002) Score tests for association between traits and haplotypes when linkage phase is ambiguous. Am J Hum Genet 70: 425–434.

6. Louis TA (1982) Finding the observed information matrix when using the EM algorithm. Journal of the Royal Statistical Society Series B (Methodological) 44: 226–233.

7. Sasieni PD (1997) From genotypes to genes: doubling the sample size. Biometrics 53: 1253–1261.

8. Devlin B, Roeder K (1999) Genomic control for association studies. Biometrics 55: 997–1004.

9. Purcell S, Neale B, Todd-Brown K, Thomas L, Ferreira MAR, et al. (2007) PLINK: a tool set for whole-genome association and population-based linkage analyses. Am J Hum Genet 81: 559–575.
